# Neutralization of Acidic Wastewater from a Steel Plant by Using CaO-Containing Waste Materials from Pulp and Paper Industries

**DOI:** 10.3390/ma14102653

**Published:** 2021-05-18

**Authors:** Tova Jarnerud, Andrey V. Karasev, Pär G. Jönsson

**Affiliations:** KTH Royal Institute of Technology, SE-100 44 Stockholm, Sweden; karasev@kth.se (A.V.K.); parj@kth.se (P.G.J.)

**Keywords:** reusing of wastes, secondary lime, neutralization, reduce landfill, acidic wastewater treatment, sustainable production

## Abstract

In this study, CaO-containing wastes from pulp and paper industries such as fly ash (FA) and calcined lime mud (LM) were utilized to neutralize and purify acidic wastewaters from the pickling processes in steel mills. The investigations were conducted by laboratory scale trials using four different batches of wastewaters and additions of two types of CaO-containing waste materials. Primary lime (PL), which is usually used for the neutralization, was also tested in the same experimental set up in the sake of comparison. The results show that these secondary lime sources can effectively increase the pH of the acidic wastewaters as good as the commonly used primary lime. Therefore, these secondary lime sources could be potential candidates for application in neutralization processes of industrial acidic wastewater treatment. Moreover, concentrations of metals (such as Cr, Fe, Ni, Mo and Zn) can decrease dramatically after neutralization by using secondary lime. The LM has a purification effect from the given metals, similar to the PL. Application of fly ash and calcined lime mud as neutralizing agents can reduce the amount of waste from pulp and paper mills sent to landfill and decrease the need for nature lime materials in the steel industry.

## 1. Introduction

The production chain of stainless steel is made up of a series of steps, where the steel undergoes various treatments to reach the desired material properties and surface qualities. During treatments at high temperatures, such as hot rolling and annealing, alloying metals from the steel matrix diffuses to the surface, react with the surrounding air oxygen and form oxide layers on steel surface. Underneath the oxide layers, some chromium depleted zones exist since the chromium diffused to the surface. These zones have lower corrosion resistances and strengths [1]. In order to remove these chromium depleted zones, a pickling process can be applied. Pickling is a chemical cleaning process that is commonly used in various steelmaking processes in a steel mill for removal of impurities (such as contaminants, corrosion products or scale) from a steel surface. The treatment aims to make the material more receptive to further processing or use. The pickle liquor usually contains strong acids such as sulfuric acid (H_2_SO_4_) and hydrochloric acid (HCl) [2]. After treating the steel in a pickling bath, the steel is rinsed with water. After rinsing, the wastewater contains high amounts of acids, and thereby has a low pH value. Before disposing or reusing of the wastewater, it has to be neutralized by addition of base reagents to avoid harming the recipient. The neutralization process is a type of a replacement reaction: Acid + Base → Salt + Water. An acid-base reaction is a chemical reaction that involves the exchange of one or more hydrogen ions. When an acid is dissolved in water, the solution has a greater hydrogen ion activity than that of pure water. When a base is dissolved in water it can accept hydrogen ions. The neutralization process is influenced by the choice of chemicals, the dosage of chemicals, the pH value and the mixing rate [1]. The pH is a major factor in neutralization and metal removal through precipitation. The metals form insoluble hydroxides at higher pH, which may be removed from the wastewater to the sediments [3]. A pH range of 8.0–11.0 minimizes the solubilities of metal hydroxides [4]. In industrial practice, the acidic wastewater is usually neutralized with natural slaked lime (denoted as primary lime in this study) to raise the pH value up to normal neutral levels.

However, by replacing primary lime with secondary lime-containing waste materials, natural resources can be saved, waste generation can be decreased and the resource efficiency can be improved. Moreover, the usage of secondary lime will also decrease the greenhouse gas emissions, since calcination of natural limestone (mainly containing CaCO_3_) generates large amounts of CO_2_ gas (780 ton CO_2_ per ton of burnt CaO produced [5]). Furthermore, it is obvious that we, the modern society, need to redirect linear material flows towards more circular flows. For instance, the possibilities to utilize secondary lime materials (fly ash and calcined lime mud) from pulp and paper production as slag formers in electric arc furnace and argon oxygen decarburization stainless steelmaking processes have been successfully tested as is described in previous publications [6,7].

In several publications ([4,8,9,10,11]) during the last decade it was reported that steelmaking slags, waste limestone materials from the marble industry [12], and cement kiln dust [13] and other secondary lime sources can be successfully applied for neutralization of acidic wastewaters and purification of industrial waters. Lime mud and recovery boiler ash can be used to remove heavy metal contaminations from metal finishing wastewater. The residual metal concentration of chromium, copper, led and zinc decreases, as the pH level of the solution increases due to direct precipitation of heavy metals by the carbonate precipitation agents calcite and burkeite. Sediments such as metal carbonates forms when carbonite reacts with heavy metals [14].

Today, some steel mills reuse the sediments from the neutralization process as flux and some steel mills send the sediments to landfills, due to that the chemical composition makes it unsuitable for reuse [15].

**Figure 1:**
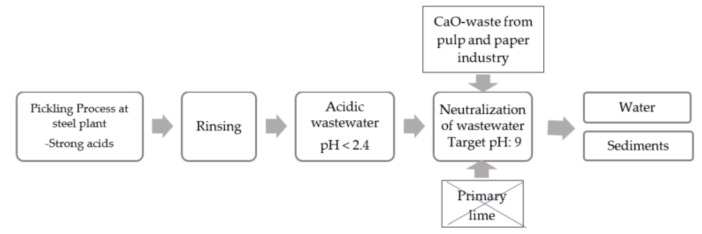
Flow from pickling to disposal of water.

In 2019, pulp and paper mills only in Sweden sent 136,000 tons of various wastes to landfills [16]. According to the European council directive 1999/31/EC on the landfill of waste, prevention, recycling and recovery of waste should be encouraged as should the use of recovered materials and energy so as to safeguard natural resources and to obviate a wasteful use of land [17]. The content of CaO-compounds in the wastes from pulp and paper industries can reach 60–90%, which is comparable or significantly larger compared to that in steelmaking slags. Therefore, it was assumed that those waste materials can also be applied for neutralization of acidic wastewater in steelmaking industries. A simplified flow scheme of the pickling wastewater is shown in Figure 1.

This study focused on investigating the possibilities to use some different CaO-containing wastes obtained from pulp and paper industries to replace primary lime in the neutralization process. Furthermore, the efficiency of neutralization of the acidic wastewater was evaluated as a function of the chemical compositions of the neutralized waters and added wastes.

## 2. Experimental

The main aim of the experimental trials was to determine the proper amount of different secondary lime materials and method of addition to raise the “potential of hydrogen” (pH) of the acidic wastewater to a value of 9 during a maximum process time of 30 min. The initial pH value of the investigated acidic wastewater was in the interval of 1.3 to 2.4. Though the pH of regular water has a value of 7, in this study, the aim was to raise the pH value to 9 since the absorption of some elements (such as Ni) from the reactants are maximized at high pH levels [8]. Furthermore, flocculants added in industrial applications will significantly lower the pH of the neutralized water.

### 2.1. Materials

**Figure 2:**
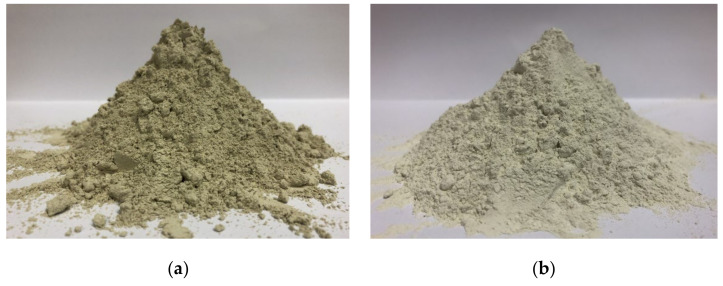
Typical photos of (**a**) fly ash powder, and (**b**) calcined lime mud powder.

In this study, two types of CaO compounds containing wastes from pulp and paper industries (so-called secondary lime sources) were used in the experimental trials, namely fly ash (FA) (Stora Enso, Hyltebruk, Sweden) and calcined lime mud (LM) (SCA, Obbola, Sweden). The FA is formed by combustion of internal and external fuels (sludge from the recycled paper and wood fuels) and LM consists of the excess chemicals from the recovery boiler in a pulp and paper mill. Moreover, an industrial primary lime (PL), which is usually applied for neutralization process in steelmaking plants, was also used in the experimental trials for sake of comparison. The main components of the lime-containing materials are given in Table 1. The LM has almost similar CaO content (90.6 wt%) as the PL (95.2 wt%). The FA has lower CaO compound contents (61.5%). Moreover, not all of the detected CaO is free CaO, since calcium silicate (Ca_2_SiO_4_) and gehlenite (Ca_2_Al_2_SiO_7_) are also present in the FA [7]. These secondary lime materials and the primary lime were used in form of powders, as delivered from the pulp and paper mills and from a steel mill. Typical photographs of the powder materials used for experimental trials are shown in Figure 2. Moreover, for some trials, the powders were additionally calcined at 1050 °C for 60 min to enable a comparison of their efficiency as neutralizing agents. Four batches of different acidic wastewaters (AWW, BWW, CWW and DWW) from two various steelmaking plants were used in the trials of this study. It should be pointed out that the acidic wastewaters from steelmaking plants are mixtures of different acids and technological waters in various combinations, depending on pickling processes by production of different steel grades. As a result, the chemical composition and pH level of each wastewater can significantly vary from batch to batch. In this study, the pH of the given acidic wastewaters equals on average to 1.92 ± 0.14 for AWW, 2.08 ± 0.14 for BWW, 1.53 ± 0.15 for CWW and 2.21 ± 0.11 for DWW.

### 2.2. Method

**Figure 3:**
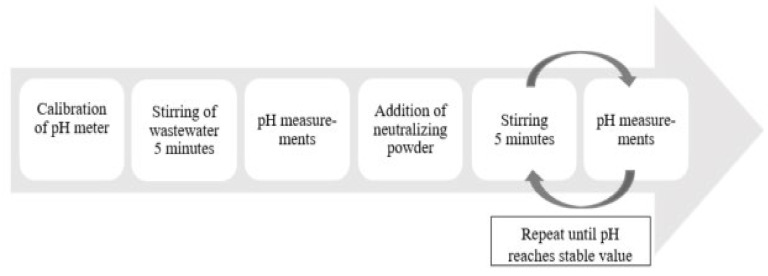
Workflow for neutralization experiments.

Acidic wastewater (210 mL) was poured into a glass beaker and stirred during 5 min by magnetic stirring to obtain a homogenization of the liquid. Then, the stirring was stopped, and the pH value of the wastewater was measured. The chosen amount of powder of secondary or primary lime was added into the beaker containing wastewater. Thereafter, the mixture was stirred for 5 min between the stops for measurements of the pH value of the solution. The pH value did not increase during the stirring stops for measurement. Therefore, only the stirring time was taken into account. The last two steps of the procedure were repeated until reaching almost constant values of pH, as illustrated in Figure 3, or until a pH value of 9 was reached.

The stirring device was run at approximately a constant speed of 500 RPM (in the span 470–530 RPM) for most trials, but stirring at speeds of ~200 and ~1000 RPM were also tested in some trials. The pH measurements were conducted by using a VWR pHenomenal IS 2100L pH-meter (VWR International, Germany). Every time the pH value was checked, three measurements were completed before a mean value was calculated. The pH-meter was calibrated by using standard technical buffers (pH 4.00, 7.00 and 10.00) every day before the experiments were started, and after three experiments had been carried out. It was also checked after a completed set of experiments. In total, 88 neutralizing experiments were made of which 40 were with FA, 26 with LM and 22 with PL.

## 3. Results and Discussion

### 3.1. Repeatability of pH Measurements

It was found that the average standard deviations (σ) for three values of pH measured in solution at the same stage of the experiment can vary between 0.1 and 0.3, which corresponds approximately to 1–3% of measured pH values in most trials. Therefore, the repeatability of the pH measurements in the given experimental trials are considered to be reliable in this study.

**Figure 4:**
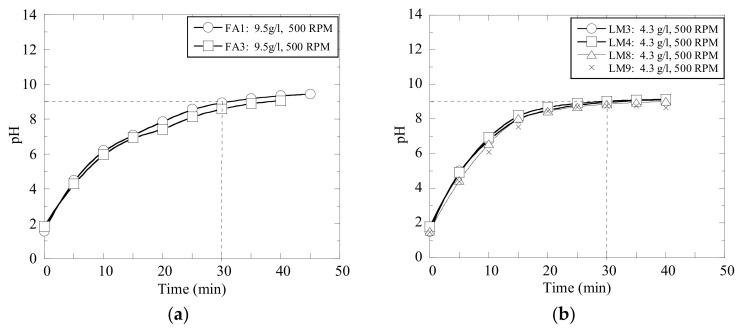
Repeatability of several experiments with (**a**) fly ash and (**b**) calcined lime mud.

Moreover, the repeatability of the pH measurements was evaluated in several experimental trials, which were carried out with the same materials and at the same given conditions. The results obtained in trials with an addition of 9.5 g of fly ash (FA) and 4.3 g of lime mud (LM) per liter of wastewater C are shown in Figure 4. It can be seen that the pH levels of the solutions consisting of the same type of wastewater and same type and ratio of neutralizing powder develop consistently, if the solution is stirred with same intensity.

### 3.2. Effect of Stirring Rate on Efficiency of the Neutralization Process

**Figure 5:**
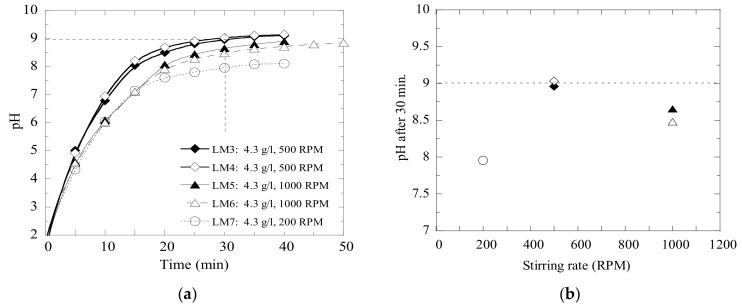
Effect of different stirring intensities (200, 500 and 1000 RPM) on the pH values. (**a**) the neutralization process and (**b**) the reached pH value after 30 min of stirring at different stirring intensities.

In order to keep the cost, and amount of sediments, at the lowest levels possible and still meet the required neutralizing rate, a ratio of neutralizer and wastewater for the prevailing circumstances needs to be established. It was found that the efficiency of the neutralization process depends significantly on the stirring intensity, as shown in Figure 5. In these trials, the same amount of lime mud powder (4.3 g/L) was added into wastewater C.

It was found that the most efficient results were obtained when using the stirring rate of 500 RPM. In this case, the pH value 9 was reached after 25 min of stirring. The least efficient process was obtained when using the lowest stirring intensity (200 RPM), as can be seen in Figure 5. Finally, the batch stirred with a 1000 RPM speed reached a pH value of 8.8 after 45 min of stirring. However, the pH value did not reach the required pH level 9 during 30 min. A similar tendency was found by Zinck and Aube [18] by evaluation of the effect of a mixing process on neutralization and cleanliness of wastewaters. They reported that the conditions of formation and growth of precipitated particles are less productive with an increased mixing rate more than optimum value. Thus, all experimental trials discussed below were carried out using the same stirring rate of 500 RPM.

### 3.3. Ratio of Required Neutralizer and Wastewater

**Figure 6:**
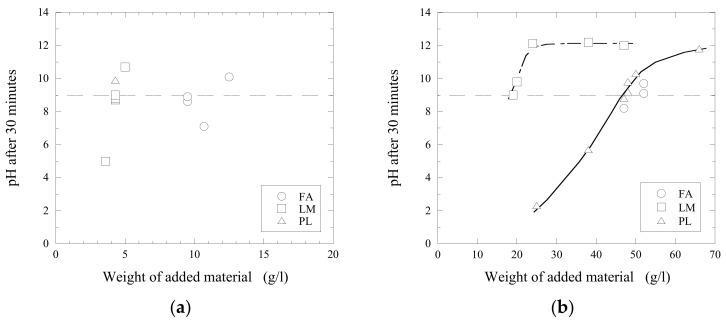
Neutralization effects obtained 30 min after the addition of fly ash (FA), lime mud (LM) and primary lime (PL) in to (**a**) C and (**b**) D acidic wastewaters.

A process of increasing the pH value of acidic wastewaters by addition of CaO-containing materials is defined in this study as a neutralization process. As can be seen in Figure 6, various wastes from pulp and paper industries have different reactivities (defined as a possibility of added material to increase the pH value of wastewater) with acidic wastewater. It is obvious that the obtained pH value increases with an increased amount of added CaO-containing materials. According to the requirements of industrial technological processes, the neutralization of wastewaters should be mostly finished during 30 min after the addition of reagents. The final required value of pH should be equal to 9, because the following additions of industrial flocculants significantly decrease the final pH value of the water.

Figure 6 shows a neutralization effect obtained in acidic wastewaters C and D after the additions of different amounts of fly ash (FA), calcined lime mud (LM) and primary lime (PL) after 30 min of stirring (at 500 RPM). It can be seen that the neutralization of wastewater C up to the given value of pH = 9 required approximately a 2.2 times larger amount of fly ash (9.5 g/L) compared to that of calcined lime mud (4.3 g/L). This can be explained by the significantly higher concentration of CaO in the LM compared to that in FA, as given in Table 1. Moreover, the original FA and PL powders consist of various complex CaO-containing components (such as CaCO_3_ and CaO-SiO_2_-Al_2_O_3_), which can be more stable and resistant in contact with acidic water compared to pure CaO. The content of such stable components can significantly decrease the reactivity of the investigated materials. A similar effect was observed for wastewater D: The required weight of FA is almost 2.7 times larger than that of LM. Even though FA contains less free CaO than LM and PL, it contains 1.8 times more MgO than PL, and 3 times more than LM. MgO reacts with water to form magnesium hydroxide (Mg(OH)_2_), which is known for its acid neutralizing capability [13].

Moreover, it was found that a further increase of added material (~24 g/L of LM and ~66 g/L of PL in D-wastewater) larger than some specific value did not promote an increase of the obtained pH value larger than ~12–12.3, as shown in Figure 6b.

**Figure 7:**
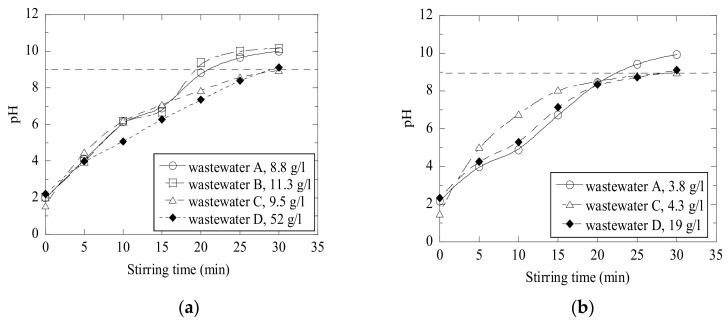
Neutralization of (**a**) wastewater A-D using fly ash and (**b**) wastewater A, C and D using calcined lime mud.

It should be pointed out that an effect of the same amount of added waste material on the pH values of different wastewaters can vary significantly. In Figure 7, some results are plotted to compare the amounts of FA and LM needed to neutralize the different batches of wastewaters. It can be seen that wastewater D requires significantly more neutralizers than the other batches of wastewater (for FA 52 g/L in the D-wastewater compared to 9–11 g/L in other wastewaters and for LM 19 g/L in the D-wastewater compared to 4 g/L in the A- and C-wastewaters). Moreover, it was found that the C-wastewater requires only 4 g/L of primary lime, while the D-wastewater requires about 48 g/L of PL to reach similar results. Specifically, this amount is 12 times higher.

**Figure 8:**
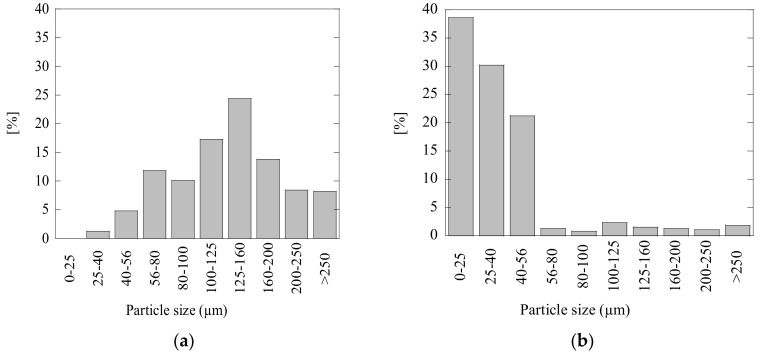
Particle-size distribution for (**a**) fly ash powder and (**b**) calcined lime mud powder used in this study.

It is important to note that the neutralization effect of acidic wastewaters can significantly depend on both compositions and other characteristics of the added powder materials (such as size distribution of particles and moisture). For instance, small sizes of particles promote faster neutralization reactions. The particle size distributions (PSD) of FA and LM powders, which were achieved by a sieve shaker range analysis using a Retch AS200 Tap, are shown in Figure 8. It can be seen that the particles of LM (<56 µm) used in the present study are much smaller than those of FA powder (40~250 µm), which can also promote a higher efficiency of the LM compared to the FA due to the higher surface/volume ratio.

**Figure 9:**
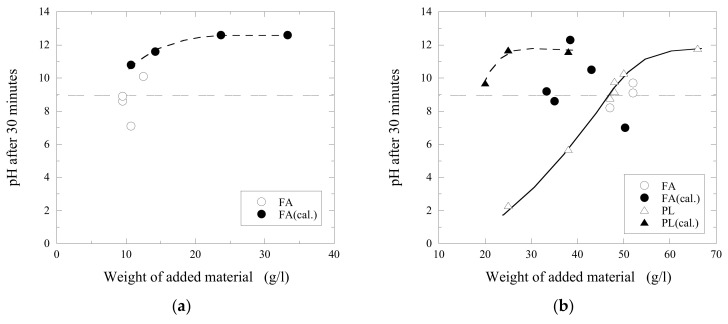
Neutralization effects obtained after 30 min after the addition of fly ash (FA) and primary lime (PL) powders without and with additional calcination in to (**a**) C and (**b**) D acidic wastewater.

In order to evaluate effect of moisture in powder materials on the neutralization process, the original powders of fly ash and primary lime were additionally calcined in a laboratory muffle furnace at 1050 °C for 60 min. Neutralization effects obtained 30 min after the addition of fly ash and primary lime powders without and with additional calcination (open and filled marks, respectively) into C and D acidic wastewaters are shown in Figure 9. By calcination of FA and PL, volatile matter and moisture are removed from the powders by thermal degradation and vaporization. This, in turn, leads to a higher concentration of substances being active in the neutralization process. It is clear that the amount of powder can be decreased significantly for both fly ash and primary lime when it is calcined. For instance, by using an additional calcination, the amount of added powder can be decreased by ~1.6 time for FA (from 52 up to 33 g/L) and 2.4 time for PL (from 48 to 20 g/L) to reach a similar pH value of approximately 9 in the D-wastewater.

**Figure 10:**
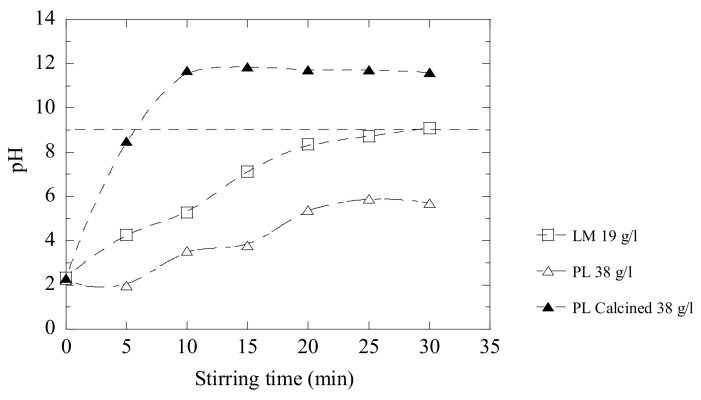
Neutralization effects obtained after the addition of LM, PL and calcined PL into D-acidic wastewater.

In Figure 10, it can be seen that LM 19 g/L reaches a pH value of 9 in 30 min, and the original PL powder at 38 g/L only reaches a pH value of 5.7 during the same time. When the PL powder was calcined prior to the trials, the efficiency is improved drastically and the maximum pH value (~12 pH) is reached in less than 10 min by using the same amount of powder. However, the calcination of the materials means of course an additional technological process and cost. Thus, this needs to be considered in the future.

### 3.4. Purification of Acidic Wastewaters During Neutralization Process

The cleaning of the wastewater is important, and it goes hand in hand with the neutralization. A variety of chemical and physical means are generally accomplished to remove heavy metals from wastewaters. Hydroxide precipitation, ion exchange, adsorption and membrane processes are some examples of treatments. Chemical precipitation is by far the most widely used process to remove heavy metals from wastewaters in the industry [19]. Neutralization and precipitation are double displacement reactions. The principle of neutralization of acidic waters by using lime lies in the insolubility of heavy metals for alkaline conditions, resulting in precipitates of hydroxides of these metals. The first step of the neutralization process is the lime dissolution. Here, the lime reacts with water and is then dissolved to increase the pH value, which can be illustrated by the following reactions:

CaO + H_2_O → Ca(OH)_2_ (1)

Ca(OH)_2_ → Ca^2+^ + 2OH^−^ (2)

When the pH value has increased, the hydroxide ions precipitate the metals. The precipitation reaction is shown with Zn as example [20]:

Zn^2+^ + 2OH^−^ → Zn(OH)_2_ (3)

In this study, the concentrations of some metals (such as Cr, Fe, Ni, Zn and Mo) were determined in the C- and D-wastewaters (CWW and DWW, respectively) before and after neutralization by addition of the FA, LM and PL. The element determination was carried out by using conventional optical emission spectrophotometers inductively coupled plasma (OES-ICP) using a SPECTRO ARCO AES-ICP model: FHX in an industrial laboratory. The obtained results are given in Table 2.

First of all, it can be seen that the DWW contained significantly larger concentrations of these metal elements compared to those in the CWW. More specifically, the concentrations of metals in D-wastewater are about 2.9–3.9 times larger with respect to the elements Cr, Fe and Mo, ~6.1 times for Ni and more than 100 times for Zn. Furthermore, it was found that the concentrations of these metals in the wastewater decreased dramatically (except for Zn) during the neutralization process in all trials when using of PL as well as LM and FA. For instance, the concentrations of Cr after neutralization decreased approximately 450–1550 times in the CWW and 1800–20,600 times in the DWW experiments. It should be pointed out that the reduction of concentrations of the given metals during neutralization of DWW is 4–60 times larger (depending on the metal) compared to the CWW results. It may be a reason why D-wastewater needs significantly larger amount of added materials for neutralization up to similar pH level.

Moreover, it can be seen that the LM has a purification effect in the acidic wastewaters from the given metals similar to the PL, while the FA has lower purification effect compared to the LM and PL.

Based on obtained results, it can be concluded that the fly ash and lime mud, which were obtained as CaO-containing wastes from pulp and paper industry, can be successfully applied instead of the common primary lime for neutralization of acidic wastewaters in steelmaking. It can promote to decrease a problem of landfill of wastes from pulp and paper industries and to reduce mining of natural limestone, which is used for production of primary lime by a calcination process generating large amount of CO_2_ gas.

## 4. Conclusions

Two types of CaO-containing waste materials (calcined lime mud and fly ash) from pulp and paper mills were tested as secondary lime sources for the neutralization of four batches of industrial acidic wastewaters (AWW, BWW, CWW and DWW) after the pickling process in steel production of two steel plants. The results obtained in the laboratory scale trials can be summarized as follows:

1. The secondary lime (in the form of fly ash (FA) and calcined lime mud (LM) from pulp and paper industry) can be successfully used instead of natural primary lime (PL) for neutralization of industrial acidic wastewaters in steelmaking plants;
2. A stirring rate of acidic wastewater with added CaO-contained materials was selected as 500 RPM, which showed better kinetic of neutralization process in the given experiments;
3. The neutralization of industrial acidic wastewaters up to the given pH value of 9 during 30 min required about 2.2–2.7 times less LM compared to that of FA. It can be explained by the significantly higher concentration of CaO in the LM and much smaller particle sizes compared to that in FA. Similar amounts of fly ash and primary lime is needed to reach the same efficiency of the neutralization process;
4. Additional calcination of PL and FA powders (at 1050 °C for 60 min) can considerably increase the neutralization effect of acidic wastewaters. For instance, the amount of added FA and PL can be decreased approximately 1.6 and 2.4 times for FA and PL, respectively, to reach a pH value of 9 in the D-wastewater;
5. Concentrations of metals (such as Cr, Fe, Ni, Mo and Zn) in steelmaking acidic wastewaters decrease dramatically after neutralization by using secondary lime. The LM has a purification effect in the acidic wastewaters from the given metals similar to the PL, while the FA has lower purification effect compared to the LM and PL.

## Figures and Tables

**Table 1:** Contents of main components in the lime-containing materials, and primary lime as reference (wt%).

Materials	CaO	SiO_2_	Al_2_O_3_	Fe_2_O_3_	K_2_O	Na_2_O	MgO	P_2_O_5_	S	Others
Fly ash (FA)	61.5	15.8	8.90	0.60	0.07	0.08	3.10	0.26	0.40	9.29
Calcined lime mud (LM)	90.6	0.18	0.07	0.04	0.10	0.48	1.05	0.75	0.11	6.62
Primary lime (PL)	95.2	1.00	0.50	0.20	0.04	0.04	1.70	0.01	0.08	1.23

**Table 2:** Results from chemical composition determinations of some metals in wastewater C and D as received, and after the addition of FA, LM and PL.

Sample	Added Neutralizer (g/L)	pH After 30 min	Cr (mg/L)	Fe (mg/L)	Ni (mg/L)	Zn (mg/L)	Mo (mg/L)
CWW Before neutralization	0		202	609	179	<0.01	40.1
C + FA *	33	12.6 in 5 min	0.13	0.02	<0.01	0.04	3.48
C + LM	4.3	8.7	0.45	1.40	1.20	0.01	6.93
C + PL	4.3	9.9	0.43	0.53	0.29	<0.01	15.7
DWW Before neutralization	0		618	2350	1084	1.5	117
D + FA	52	9.1	0.08	0.10	0.04	<0.01	5.06
D + FA *	33	8.6	0.34	0.35	0.28	<0.01	4.66
D + LM	19	9.1	0.10	0.54	1.67	0.01	1.06
D + PL	47	8.8	0.11	0.45	156	0.47	0.17
D + PL	48	9.2	0.03	0.12	0.15	0.01	2.46

* powder used after additional calcination at 1050 °C during 60 min.

## Data Availability

Data sharing is not applicable to this article.
